# A Six-Month Randomized Controlled Trial of Whole Soy and Isoflavones Daidzein on Body Composition in Equol-Producing Postmenopausal Women with Prehypertension

**DOI:** 10.1155/2013/359763

**Published:** 2013-08-01

**Authors:** Zhao-min Liu, Suzanne C. Ho, Yu-ming Chen, Jean Woo

**Affiliations:** ^1^Department of Medicine & Therapeutics, The Chinese University of Hong Kong, Hong Kong; ^2^Division of Epidemiology, The Jockey Club School of Public Health and Primary Care, The Chinese University of Hong Kong, Hong Kong; ^3^Department of Medical Statistics and Epidemiology, School of Public Health, Sun Yat-sen University, Guangzhou 510080, China; ^4^Division of Geriatrics, Department of Medicine & Therapeutics, The Chinese University of Hong Kong, Hong Kong

## Abstract

*Objectives*. This paper reported the effects of commonly used whole soy foods (soy flour) and purified daidzein (one of the major isoflavones and the precursor of equol) on changes in anthropometric measurements and body composition in a 6-month double-blind, randomized, placebo-controlled trial among prehypertensive postmenopausal women who are also equol producers. *Methods*. 270 eligible women were randomized to either one of the three treatments: 40 g soy flour (whole soy group), 40 g low-fat milk powder + 63 mg daidzein (daidzein group), or 40 g low-fat milk powder (placebo group) daily each for 6 months. Anthropometric indicators and body composition were measured before and after intervention. *Results*. 253 subjects completed the study with good compliance. Urinary isoflavones levels suggested good compliance of subjects with supplementation. Whole soy and purified daidzein had no significant effect on body weight, body mass index (BMI), waist and hip circumferences, waist to hip ratio (WHR), body fat percentage, fat mass, and free fat mass. Conclusion. Six-month consumption of whole soy and purified daidzein at provided dosage had no improvement on body weight and composition compared with isocaloric milk placebo among prehypertensive equol-producing postmenopausal women. This trial is registered with ClinicalTrials.gov NCT01270737.

## 1. Introduction 

Overweight and obesity are important clinical and public health burdens worldwide affecting more than 30% of adult population [1]. Aging- and menopause-induced estrogen deficiency results in an increase in body weight or abdominal fat and a decrease in lean mass which contribute to an elevated risk of cardiovascular diseases (CVD) and other metabolic disorders [2, 3]. Diet therapy has the lowest side effects for the prevention and management of postmenopausal obesity relative to hormone replacement therapy [4]. Soy is a traditional Asian diet and also a rich source of plant protein, unsaturated fat, dietary fiber, isoflavones (one major phytoestrogen), saponin, and so forth with a potential role on fat mass reduction and weight control. 

Laboratory and animal studies are generally supportive for soy and/or its components when isocaloric diets are administered to promote weight and fat loss by increasing energy utilization [5], reducing fat accumulation [6], and promoting a select loss of visceral adipose tissue [7] with more benefits than those of casein or other animal foods [8, 9]. Several observational studies [10–12], but not all [13], have reported that soy or isoflavone correlated with lower body mass index (BMI), waist circumference (WC), or body fat mass. However, clinical trials indicated inconsistent findings. Some studies reported a favorable effect with soy protein or isoflavones intake on body weight and fat mass reduction [14–17], while others not [18–21]. The differences in population characteristics, types of soy products, duration of intervention, methods for outcome measures, capacity for isoflavones metabolization may explain the inconsistent findings among studies. 

 The major soy isoflavones include genistein and daidzein. There is increasing evidence that clinical efficacy of isoflavones in humans depends on the ability to produce equol, a microbial metabolite of daidzein that is more estrogenic and antioxidant than daidzein [22, 23]. As only 20–50% human adults can produce equol after ingestion of soy foods, equol production might be an important modifier of the effects of soy [24]. To date, no RCT has tested the independent effects of daidzein on body weight and composition and studies specifically designed among equol producers are limited. In addition, most of clinical trials investigating the effect of soy on body composition were with energy restriction; however, high intensity weight-loss may decrease the likelihood of detecting significant differences between experimental arms. 

Current evidence on soy and health benefits favors equol producers rather than nonproducers [25, 26] and whole soy foods (less processed soy products such as soy nuts, soy flour, soy milk, and tofu) rather than isolated soy component [27]. Also, increasing interests have been on directly comparing the effects of isolated dietary components and the food from which these constituents are derived [28]. Thus, this paper reported the effects of whole soy and purified daidzein without energy restriction on changes in anthropometric measurements and body composition. This is the part of results of our 6-month double-blind, randomized, placebo controlled trial examining the effect of whole soy and daidzein on blood pressure and cardiovascular risks among 270 prehypertensive postmenopausal Chinese women who are also equol producers. We hypothesized that whole soy intake or purified daidzein would improve body composition compared with milk placebo.

## 2. Methods

### 2.1. Subjects Recruitment

The study was a randomized, double-blind, placebo-controlled trial. The enrollment of participants was conducted from December 2010 to January 2012 in local community through advertisements in newspaper. Participants were initially screened by telephone interview and their equol-producing phenotypes were then confirmed by consecutive intake of 63 mg daidzein for 7 days and collection of 24 h urine for isoflavones testing. Written informed consent was obtained from each participant prior to enrolment. The institutional review boards of the Chinese University of Hong Kong approved the study protocol. 

The eligible subjects were Hong Kong Chinese women aged 48–65 y; at least 1 year menopausal; mean SBP above 120 mmHg, DBP above 80 mmHg, or both; equol producers which were defined as 24-hour urinary *log*_10_ *S*-equol/daidzein ratio greater than −1.75 after daidzein challenge [29]. Subjects were excluded if they were currently or in preceding 6 months taking antihypertensive, hypoglycemic, or weight reduction agents or on hormone therapy; had a history of stroke, coronary heart diseases, untreated thyroid diseases, severe liver, and renal dysfunction; have breast, uterine, or ovarian cancer or other malignancies in previous 10 years; on prescribed weight control or vegetarian diet; with known soy or milk allergy. 

### 2.2. Study Power

With a planned number of subjects of 90 in each arm (270 subjects in total) and given the current SD of changes in body weight and fatness percentage, we would have 80% power to find a 0.75 kg change in the body weight (SD of change 2.2 kg) and 0.72% in the change of body fat percentage (SD of change 2.1%), based on a convention assumption of *α* level 0.05 (for a 2-side *t*-test) and allowing for 10% withdrawal rate.

### 2.3. Randomization

A block randomization was used for subject assignment in random block sizes of 6, 9, and 12. The 270 continuous serial numbers were divided into 30 blocks. A list of random numbers was computer generated and each random number corresponds with 1 of the 3 possible interventions. 270 serial numbers were labeled on the identically looking boxes of supplements by personnel not involved in the trial and assigned to eligible subjects according to the sequence of their visits. All the research staff, technicians, and subjects were blinded to the treatment codes. To assess blinding, we asked the participants which group they thought they had been assigned to at the end of the trial. The rates of correct estimation of allocation were 7.8%, 7.8%, and 6.5% in whole soy, daidzein, and placebo groups, respectively (*P* = 0.947), indicating the blinding was effective.

### 2.4. Intervention and Supplement Preparation

Eligible participants were randomized to one of the 3 groups: 40 g soy flour (whole soy group), 40 g low-fat milk powder + 63 mg daidzein (daidzein group), or 40 g low-fat milk powder (placebo group) daily each for 6 month. The 3 kinds of supplements were processed into beverage powder with similar color, odor, and major nutrients profile by addition of minerals, starch, or other food additives.  Table 1 indicated the nutrient profiles of the 3 supplements. The daily dose was filled into identical looking sachets. The products were tested prior to and after packaging to ensure content uniformity. The supplements were suggested to be mixed with 300 mL of water or beverages and partially replace breakfast or snacks. Participants were asked not to take supplements containing phytoestrogen or other extracts known to affect outcome measures, minimize their intake of soy foods (≤2 servings per week), refrain from high salt diet, restrict alcohol intake (≤2 drinks per week), and maintain their usual level of physical activity. They were required to return the unconsumed sachets at each followup to assess their adherence. A monthly telephone interview was conducted during the intervention and any changes in diet, physical activity, health status, and medications were monitored by research staff.

### 2.5. Data Collection

Individual information was collected by trained interviewers by face-to-face interview based on a structured questionnaire on sociodemographic data, years since menopause, medical history, medication, dietary habits and physical activities, and so forth. Dietary intakes over intervention were evaluated by 3-day food diary which was recorded by subjects at baseline and at the end of the trial. Subjects received a 30-min training on estimation of food amounts, portion and utensil sizes. Dietary nutrients (energy, protein, total fat, calcium and soy isoflavones) were calculated based on the China Food Composition Table [30]. 

### 2.6. Anthropometric Measurements and Body Composition

Anthropometric indicators and body composition were measured at baseline and end of the trial. Body weight was measured with the subjects on light clothing without shoes by using the beam balance scales (Detecto, Clinicon Medical LTD, USA) and recorded to the nearest 0.1 kg. Height was measured in a standing position without shoes by using a wall-mounted stadiometer. Body mass index (BMI) was calculated as weight in kilograms divided by height in meters squared. Waist circumference was measured at the level halfway between the lowest rib and the ileac crest using a nonelastic tape without any pressure to body. Hip circumference was measured at the level of the greater trochanters. WHR was calculated as the ratio of waist-to-hip circumferences. All measurements were performed twice by the same research staff and the average was recorded.

 Body fat percentage (BF%), fat mass (FM), and free fat mass (FFM) were assessed by bioelectrical impedance analyzer (BIA, TBF-410-GS Tanita Body Composition Analyzer, Japan). The BF% was calculated from the impedance value as well as the preentered personal particulars including weight, height, age, and sex through a build-in software [31]. These measurements were done after 10–12 hours fasting. Subjects were requested to refrain from strenuous exercise and empty their bladders before the measurements. The coefficients of variation for repeated measures were all less than 5% for BF%, FM, and FFM. 

### 2.7. Statistical Analysis

Statistical analysis was performed with the use of SPSS 16.0 software. Results were considered significant if the two-tailed *P* value was <0.05. Baseline characteristics, dietary intake, and physical activity level were compared among the 3 study groups by analysis of variance (ANOVA). Data were analysed according to an intention-to-treat principle including all 270 subjects who were randomized. The last value carried forward was used for any missing data at followups. The changes and percentage changes at 6-month in outcome variables among the 3 study groups were compared by ANOVA. Bonferroni test was used for Post Hoc multiple comparisons. We tested the effect modification by adding interaction terms of intervention and subgroup variables to the univariate model. A further stratification analysis was conducted to examine whether the effect was differed by baseline BMI level and years since menopause. 

### 2.8. Assessment of Compliance

Compliance with treatment was assessed by counting the returned supplements for estimation of percentage of intake, food diary and 24-h urinary excretion of isoflavones at baseline and at 6 months. Urinary isoflavones were tested by high performance liquid chromatography (HPLC) [32]. Adherence was good and did not differ among the 3 groups. 

## 3. Results

**Figure 1:**
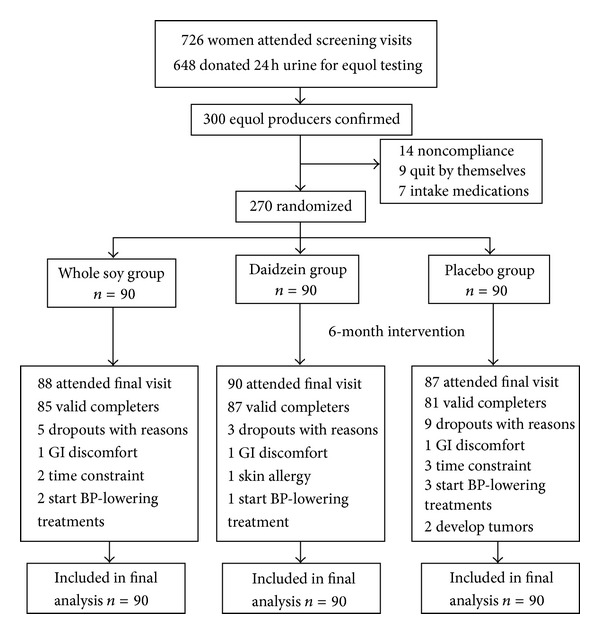
GI discomfort: gastrointestinal discomfort; BP: blood pressure; Whole soy group: intake 40 g soy flour per day; Daidzein group: intake 63 mg daidzein + 40 g low-fat milk powder per day; Placebo group: intake 40 g low-fat milk powder per day.

Overall 270 eligible women were randomized into 3 intervention arms. Seventeen (6.3%) subjects withdrew: 5 in the whole soy group, 3 in the daidzein group, and 9 in the placebo group. There was no significant difference in dropout rates between the 3 groups (*P* = 0.172). Detailed study flow and reasons of withdrawal were indicated in Figure 1. Subjects who withdrew from the study were also invited for follow-up visits with 265 subjects attending the final visit.

Baseline characteristics (Table 2) were similar among the 3 study groups in terms of age, menopausal year, BMI, SBP and DBP, education, medical history, medication, dietary habits, and physical activity. There was no significant difference in the baseline and follow-up dietary intake in total energy, protein, total fat, and soy isoflavones among the 3 study groups. 

No significant difference was observed in the 6-month change or %changes in body weight, BMI, waist and hip circumferences, WHR, body fat%, fat mass, and free fat mass among the 3 study groups (Table 3). There was no significant interaction between intervention and years since menopause, however, a significant interaction in intervention and basal body weight (*P* = 0.03). Further subgroup analysis among subjects with BMI > 24 suggested a notable reduction in BMI and WHR with milk placebo intake. 

### 3.1. Adverse Events

A total 182 adverse events were reported, approximately equally distributed among the 3 treatment phases. The most common complaints were gastrointestinal discomfort (*n* = 166), sore throat (*n* = 21), weight gain (*n* = 18), rash (*n* = 13), and feelings of breast swelling (*n* = 7, 2 in soy flour group, 3 in daidzein group, and 2 in placebo group). Two women in the daidzein group, both within 3 years since menopause, reported a 2~3 days episode of vaginal bleeding after 2 months' intervention. No recurrence and abnormalities were reported.

### 3.2. Compliance

The consumption of supplements from those provided (including dropouts) were 93.3% in the whole soy group, 94.4% in the daidzein group, and 92.9% in the placebo group throughout the trial. Ninety-nine participants were randomly selected for final 24 h urinary isoflavones testing for compliance assessment. The purpose of final urine collection was not disclosed to subjects. Urinary genistein and glycetin increased 7.0 and 4.2-fold in whole soy group, while urinary daidzein and equol increased 4.7- and 6.6-fold in daidzein group with no change in milk placebo group, indicating good compliance among participants.

## 4. Discussion

Our 6-month double-blind randomized placebo-controlled trial among prehypertensive, equol-producing postmenopausal women demonstrated that whole soy (containing 12.8 g/d soy protein) and purified daidzein (63 mg/day) had no significant effect on anthropometric parameters and body composition compared with isocaloric milk placebo. 

Although data from animal models generally support a favorable effect of soy consumption on weight and fat loss, results from clinical trials are still controversial. Our findings are in line with several previous RCTs [19, 21, 33–35] in which soy protein has no better effect on body weight and composition than milk or animal products. A 12-month RCT [34] among older postmenopausal women (60–75 y) reported that 25.6 g soy protein containing 99 mg isoflavones had no effect on BMI and WHR compared with milk protein. Women at different menopausal stages may response differently to soy supplementation; however, our subgroup analysis among women in early menopause (<4 years) did not suggest a different effect compared with women in late menopause. Another 20-week RCT [19] even reported that formula food containing milk protein is superior to that containing soy protein for reducing visceral and subcutaneous fat. A 23-week RCT [20] also indicated that whey protein but not soy protein supplementation alters body weight and composition in overweight and obese adults. Our results also indicated a trend of reduction in body weight with milk placebo intake (*P* = 0.095), at least implying that soy consumption does not have advantage over low-fat milk on weight and fat loss. Previous evidence also supported that there is no difference in satiety between dietary soy protein, other vegetable proteins, and animal protein [36, 37]. As in many other studies, we used milk product to serve as the placebo. However, milk peptides may have active biological properties relevant to weight loss and obesity [38], thus comparing two biologically active interventions (soy and milk) may blunt the potentially notable effect of soy. 

 The nonsignificant findings in our study may be explained by the fact that most of our participants were not overweight or obese at the start of the trial. The average BMI of our participants was 23.4 while 38.5% were overweight or obese (BMI ≥ 24). Subgroup analysis among BMI ≥ 24 even suggested a more apparent reduction in body weight with milk placebo intake relative to whole soy. Previous evidence among normal weight individuals is limited; however, clinical trials in obese patients did not consistently support the favorable effect of soy on body weight and composition [21, 33, 39]. In addition, our subjects consisted of women with prehypertension or hypertension who were also equol producers, a population with the most potential to benefit from soy intervention. Thus, the relative normal basal body weight cannot fully explain the absent effect of soy on weight and body fatness. 

The neutral findings could also be due to the inadequate soy protein dosage. A higher soy protein intake is hypothesized to produce more notable differences in weight loss and body composition. However, clinical trials [40–42] with soy intake higher than current study indicated inconsistent findings and no dose-response relationship reported. Although our previous RCT [42] reported a mild but significant effect with 15 g soy protein and 100 mg isoflavones on anthropometric measures relative to milk protein, a study by Moeller et al. [40] using a higher dosage of soy protein (40 g soy protein and 80.4 mg isoflavones) did not affect body fat and lean mass compared to whey protein. In the present study, we used a daily dose of 40 g soy flour which contains 12.8 g soy protein and 49 mg isoflavones representing a highly normal range of a typical soy intake in Asian countries [43]. This amount corresponds to the recommendation of the Chinese Nutrition Society of daily intake of 30–50 g soy products [44]. A higher dosage may strongly modify the dietary habits of participants and affect the compliance during a relatively long-term treatment. In addition, studies have shown that isolated soy component is not as effective as intact soy foods on improvement of cardiovascular health [45]. Moreover, Asia women with similar study age (45~65 y) have conventionally high soy food consumption, which could possibly neutralize the treatment effect of soy. To avoid possible ceiling effect, we instructed our subjects to reduce their habitual soy intake to no more than 2 servings per week. The dietary assessment at baseline and the end of trial indicated that a good dietary compliance of subjects with a reduction of average isoflavones intake decreased from 14.5 to 8.3 g/d. Thus, the soy dosage in our study seems unlikely to be the key issue of the effect of soy.

The study has several limitations. Firstly, the use of bioelectrical impedance method (BIA) for body composition measures had advantages but also limitations. Body fat should ideally be measured by the golden standards, densitometry (underwater weighing), or dual X-ray absorptiometry (DXA), but these two methods are generally expensive and impractical for epidemiologic study. BIA is a practical and good alternative [46, 47] with established validity and reliability in the assessment of body composition [48, 49]. The precision error of BIA is similar to DXA and UW [50]. BIA instrument used in our study also has limitation in differentiating regional fat from total fat mass, as evidence has shown that central adiposity is more strongly associated with CVD than peripheral adiposity [51]. However in our study the change in waist circumference (a validated indicator of intra-abdominal adipose tissue) did not differed among the 3 study arms. Secondly, milk product may not be an adequate “placebo” for investigating the effects of soy on body weight control. In addition, the increase in protein and energy intake due to supplementation may have overshadowed the treatment effect.

To our knowledge, this is the first RCT concomitantly comparing the effects of commonly used whole soy foods (soy flour) and potential active soy component of daidzein among equol-producing women, a susceptible population to benefit from soy intervention. This study was a randomized, double-blind, placebo-controlled trial with a relatively large sample size. Subjects' compliance was good and the attrition rate was only 6.3%. The study also had successful blindness efficacy. Three supplements were formulated into isocaloric powder with similar nutrient profiles. Furthermore, dietary intake and physical activity were stable across the whole intervention which was unlikely to confound the effects of soy. To better ensure participant compliance with treatment, we qualitatively measured urinary excretion of isoflavones at baseline and the end of the trial. Also, participants were instructed to restrict their usual soy intake to no more than 2 servings of soy foods per week to avoid a potential “ceiling effect” of soy. Finally, based on the number of participants who completed the intervention, this study was well powered to detect a small change in body weight and body fatness. 

## 5. Conclusion

The 6-month RCT among equol-producing postmenopausal women with prehypertension or hypertension indicates that soy consumption does not exert an advantage over low-fat milk product for weight and fat loss when prescribed at isocaloric levels. Future trials among overweight or obese patients, using more elaborate techniques to assess body composition are warranted.

## Figures and Tables

**Table 1:** Nutrient profiles of the 3 study supplements (daily dosage).

	Whole soy group	Daidzein group	Placebo group
Energy (kcal)	199.6	192.5	192.5
Protein (g)	12.8	12.7	12.7
Total fat (g/d)	7.6	6.3	6.3
Total isoflavones (mg)	49.3	63.0	0
Genist(e)in (mg)	19.4	0	0
Daidz(e)in (mg)	23.2	63.0	0
Glycit(e)in (mg)	6.7	0	0

Soy flour was purchased from Land Reclamation Dragon and King Foods Co. Ltd. (Heilongjiang, China), purified daidzein from Shanxi Sciphar Hi-Tech Industry Co. Ltd, and milk protein from Pacific Dairy Ingredients Co. Ltd. (shanghai, china). Supplements' isoflavones content was tested by high-performance liquid chromatography (HPLC).

**Table 2:** Baseline characteristics among 3 study groups.

	Whole soy group	Daidzein group	Placebo group	*P* value
	*n* = 90	*n* = 90	*n* = 90	
Age (y)	57.6 ± 5.3	57.7 ± 5.0	58.5 ± 4.7	0.464
Menopausal years (y)	9.0 ± 6.4	8.9 ± 5.7	9.1 ± 5.2	0.977
BS Total PA (MET min/d)	1251.3 ± 726.4	1218.5 ± 625.1	1134.2 ± 534.7	0.450
Job PA	476.6 ± 565.3	453.8 ± 618.7	386.9 ± 594.5	0.574
House PA	550.6 ± 571.2	568.6 ± 506.6	509.1 ± 340.9	0.700
Exercise PA	221.6 ± 200.2	191.6 ± 178.8	229.2 ± 206.3	0.403
BS dietary intake				
Energy (kcal/d)	2048.3 ± 543.2	2090.4 ± 655.25	1983.5 ± 431.6	0. 341
Protein (g/d)	88.9 ± 22.7	90.1 ± 29.4	86.1 ± 20.6	0.373
Fat (g/d)	64.9 ± 21.8	69.0 ± 22.6	63.4 ± 37.1	0.386
Isoflavones (mg/d)	14.6 ± 10.1	14.2 ± 8.5	14.5 ± 9.5	0.852
Dietary intake at 6 month (not including supplements)				
Energy (kcal/d)	1956.2 ± 475.65	2006.9 ± 560.6	1938.7 ± 391.1	0.443
Protein (g/d)	82.6 ± 21.9	80.8 ± 27.5	76.5 ± 18.4	0.368
Fat (g/d)	61.5 ± 19.8	63.6 ± 15.8	60.4 ± 23.9	0.325
Isoflavones (mg/d)	8.6 ± 6.6	8.3 ± 7.5	7.9 ± 7.2	0.714
Job status				0.128
housewife	37 (13.7%)	46 (17.0%)	54 (20.0%)	
Part-time job	26 (9.6%)	18 (6.7%)	16 (5.9%)	
Full-time job	27 (10.0%)	26 (9.6%)	20 (7.4%)	
Ever use of lipid-lowering medication	8 (7.5%)	8 (7.5%)	8 (7.5%)	0.915
Ever use of HRT	9 (3.3%)	13 (4.8%)	19 (7.0%)	0.112
Ever use of contraceptives	46 (17.0%)	39 (14.4%)	45 (16.7%)	0.528
Passive smoking	13 (4.8%)	12 (4.4%)	15 (5.6%)	0.814
Regular alcohol drinking	7 (2.6%)	8 (3.0%)	10 (3.7%)	0.738
Regular coffee drinking	34 (12.6%)	37 (13.7%)	32 (11.9%)	0.742
Regular tea drinking	73 (32.7%)	73 (32.7%)	77 (34.5%)	0.662

Data are presented as mean ± standard deviation for continuous variables or number (%) for categorical variables. ANOVA test for continuous variables and Chi-square test for categorical variables. BS indicates baseline; PA indicates physical activity; HRT indicates hormone replacement treatment; Regular drinking means drinking alcohol, tea, or coffee more than 1 time per week. METs are multiples of resting metabolic rates and an MET minute is computed by multiplying the MET score of an activity by the minutes performed. Dietary nutrient intakes were calculated mainly based on the China Food Composition Table 2002 and 2004.

**Table 3:** The effect of whole soy and purified daidzein on anthropometric measures and body composition.

	Whole soy group	Daidzein group	Placebo group	*P* values
	*n* = 90	*n* = 90	*n* = 90	
Body weight (kg)				
Baseline	56.6 ± 7.33	56.3 ± 9.18	57.7 ± 9.01	0.552
6-month	56.5 ± 7.21	57.0 ± 9.16	57.0 ± 8.45	0.901
Change	0.20 ± 1.78	0.59 ± 3.35	−0.20 ± 1.53	0.095
%change	0.399 ± 3.347	1.272 ± 6.971	−0.311 ± 2.649	0.092
Body mass index (BMI, kg/m^2^)				
Baseline	23.4 ± 2.78	23.2 ± 3.46	23.7 ± 3.34	0.552
6-month	23.5 ± 2.78	23.5 ± 3.42	23.6 ± 3.21	0.964
Change	0.163 ± 0.758	0.331 ± 1.388	0.037 ± 0.622	0.143
%change	0.746 ± 3.331	1.672 ± 7.073	0.171 ± 2.574	0.112
Waist circumference (WC, cm)				
Baseline	79.1 ± 7.70	78.1 ± 8.33	78.4 ± 8.84	0.693
6-month	78.1 ± 7.85	77.4 ± 8.41	76.9 ± 7.90	0.600
Change	−0.816 ± 2.779	−0.706 ± 2.503	−1.039 ± 2.725	0.701
%change	−0.991 ± 3.522	−0.877 ± 3.130	−1.217 ± 3.298	0.787
Hip circumference (HC, cm)				
Baseline	93.1 ± 5.88	93.1 ± 7.09	93.8 ± 7.04	0.736
6-month	93.0 ± 6.02	93.1 ± 7.10	93.1 ± 6.55	0.995
Change	−0.094 ± 1.627	0.009 ± 1.547	−0.195 ± 1.547	0.691
%change	−0.092 ± 1.792	0.019 ± 1.664	−0.194 ± 1.660	0.708
Waist and hip ratio (WHR)				
Baseline	0.848 ± 0.047	0.838 ± 0.047	0.834 ± 0.052	0.137
6-month	0.839 ± 0.044	0.830 ± 0.049	0.826 ± 0.050	0.171
Change	−0.008 ± 0.0265	−0.008 ± 0.022	−0.009 ± 0.024	0.937
%change	−0.899 ± 3.073	−0.899 ± 2.561	−1.026 ± 2.821	0.942
Body fat percentage (%)*				
Baseline	30.5 ± 5.87	30.5 ± 6.55	30.6 ± 6.41	0.992
6-month	30.7 ± 6.01	30.1 ± 6.17	30.4 ± 5.94	0.812
Change	0.159 ± 2.104	−0.382 ± 2.241	0.122 ± 2.087	0.180
%change	0.691 ± 6.805	−0.786 ± 7.378	0.507 ± 6.421	0.306
Fat mass (kg)				
Baseline	17.4 ± 5.30	17.9 ± 6.69	18.0 ± 6.56	0.831
6-month	17.6 ± 5.54	17.5 ± 6.04	17.6 ± 6.06	0.991
Change	0.233 ± 1.584	−0.310 ± 1.844	0.099 ± 1.669	0.092
%change	1.517 ± 9.440	−0.542 ± 9.400	0.550 ± 8.055	0.319
Free fat mass (kg)				
Baseline	38.6 ± 3.62	38.8 ± 4.08	39.1 ± 3.51	0.673
6-month	38.7 ± 3.23	38.9 ± 5.63	38.9 ± 3.21	0.894
Change	0.165 ± 0.956	0.121 ± 3.484	−0.129 ± 1.198	0.640
%change	0.519 ± 2.499	0.200 ± 9.867	−0.223 ± 2.796	

All values are means ± standard deviation; *P* values refer to comparison among the 3 study groups by ANOVA (analysis of variance); *Skewed variables or variables with heterogeneity in variance (Body fat%) were corrected by log transformation and reported arithmetic means.
